# Aware, motivated and striving for a ‘safe tan’: an exploratory mixed-method study of sun-protection during holidays

**DOI:** 10.1080/21642850.2017.1335205

**Published:** 2017-06-05

**Authors:** Angela M. Rodrigues, Falko F. Sniehotta, Mark A. Birch-Machin, Vera Araujo-Soares

**Affiliations:** ^a^ Institute of Health and Society, Medical Faculty, Newcastle University, Newcastle upon Tyne, UK; ^b^ Institute of Cellular Medicine, Medical Faculty, Newcastle University, Newcastle upon Tyne, UK

**Keywords:** Sun-safety, tanning, skin cancer, public health, interview

## Abstract

**Background:** This article presents an exploratory study, aiming to explore the correspondence between knowledge, motivation and sun-protection practices during holidays.

**Methods:** Seventeen participants aged 21–62 years old, recruited from community settings took part in individual face-to-face semi-structured interviews, completed sun sensitivity questions and an objective assessment of sunscreen use. Holidaymakers’ knowledge about sun-safe messages, intentions and perceptions of barriers and facilitators for sun-protection were assessed. Qualitative data were analysed using thematic analysis and integrated with quantitative data, using a pragmatic theory-informed approach to synthesise the findings.

**Results:** Participants were well informed about sun-safe messages, highly motivated to protect themselves from solar UV radiation (UVR) and they perceived themselves as well protected. However, they did not seem to use effective protective practices. Sunscreen was the preferred method of sun-protection, but most participants used considerably less than the recommended amount and significantly overestimated the amount of time they could be safely exposed. Seeking shade was the least used method of sun-protection and covering-up strategies were mostly implemented as a partial protection (i.e. hats or sunglasses). The desire to reach an optimal balance between getting a tan and using sun-protection to avoid sunburns was preeminent. Several additional barriers and facilitators for sun-protection were identified.

**Conclusions:** Holidaymakers might have a false sense of security when it comes to sun-exposure. They are aware of the need to protect from solar UVR, but the motive for a safe tan, the overreliance on sunscreen, the overestimation of the safe sun-exposure time for their skin type and the insufficient application of sunscreen leaves holidaymakers motivated to protect their skin at significant risk of overexposure, sunburn and skin cancer. Public health messages need to address how to implement effective sun-safe strategies.

## Introduction

### Background

Skin cancers have been increasing worldwide over the past decades, in particular among Caucasian populations. Incidence rates are as high as 34.9 cases in 100,000 in Australia and around 14 in 100,000 in both the USA and UK (Ferlay et al., 2012). Skin cancer results from a combination of non-modifiable risk factors (i.e. skin phenotype) and lifestyle factors (e.g. exposure to ultraviolet radiation [UVR]). Overexposure to UVR and history of sunburns (often obtained through holidays in sunny destinations) are the main risk factors for skin cancer (Parkin, Mesher, & Sasieni, 2011).

The public health messages for skin cancer prevention emphasise the importance of reducing sun-exposure and sunburns by encouraging people to seek shade during peak hours (midday) and wearing protective clothes (National Institute for Health and Clinical Excellence [NICE], 2011b). They also recommend applying a broad-spectrum sunscreen of SPF 15+ (Cancer Research UK, 2009b; The British Association of Dermatologists, 2013; World Health Organisation, 2014) but emphasise that sunscreen should not be the first line of defence (Autier et al., 1998; Lazovich et al., 2011; Linos et al., 2011). Interventions promoting these sun-protection messages are effective (Rodrigues, Sniehotta, & Araujo-Soares, 2013; Saraiya et al., 2004) and various national public health campaigns have actively promoted safer sun behaviours over the past decade (National Institute for Health and Clinical Excellence (NICE), 2011a). For example, the Anti-Cancer Council of Victoria launched in 1980 a large-scale campaign branded ‘Slip! Slop! Slap!’ to reduce individuals’ sun-exposure by advising the population to slip on a shirt, slop on some sunscreen, and slap on a hat (Montague, Borland, & Sinclair, 2001). Epidemiological evidence suggests that these messages have been effective in slowing melanoma incidence rates for younger age groups (Sinclair & Foley, 2009). In the UK, the SunSmart campaign was launched in 2003 and it represents the national campaign for skin cancer prevention conducted by Cancer Research UK (Cancer Research UK, 2011). Holidaymakers are not currently the primary focus of the SunSmart campaign in the UK.

Some behaviour theories provide insight into mechanisms of behaviour enactment and change. Key change mechanisms identified by some of these models and theories can support the development of skin cancer prevention campaigns. Skin cancer prevention messages need to: (a) provide members of the public with the necessary level of knowledge and awareness; (b) enable people to consider barriers and facilitators of adopting sun-safe behaviours; (c) enable people to form an intention to be sun-safe and (d) support people to enact this intention in line with current guidelines for sun-protection (Rogers, 1975; Schwarzer, 1992; Weinstein, 1988).

Various surveys show that the awareness of the need to be sun-safe, intentions to prevent sunburns (Allom, Mullan, & Sebastian, 2013; Araujo-Soares, Rodrigues, Presseau, & Sniehotta, 2013; Geller, Cantor, et al., 2002; Geller, Colditz, et al., 2002; Heckman & Coups, 2011), as well as self-reported sun-protection behaviours are increasing over time (Cancer Research UK, 2009; Centers for Disease Control and Prevention, 2012). However, contrary to the focus of public health messages of seeking shade and using protective clothing, the most frequently reported sun-protection behaviour in population surveys is the use of sunscreen. For example, results from a large survey in the UK showed that 83.9% used sunscreen as a form of sun-protection, whilst only 41.0% of participants reported staying in the shade, 15.1% limiting the time spent in the sun and 39.9% using covering-up strategies (Cancer Research UK, 2009a).

The overreliance on sunscreen as a mode of protection increases the vulnerability for sunburns in the population. Sunscreen use is a complex preventive behaviour as its protective features depend both on the amount of sunscreen used, the time of subsequent sun-exposure relative to an individuals’ skin type (Chesnut & Kim, 2012; Diffey, 2009; Green, Williams, Logan, & Strutton, 2011) and other sustainability features such as water resistance. The general guideline for sunscreen application is to use 2 mg of sunscreen per cm^2^ of skin surface (The British Association of Dermatologists, 2013). This equals about 35 grams of sunscreen (approximately a third of a 100 ml sunscreen bottle) for a full body application in a typical adult per application, but evidence suggests that most users apply insufficient amounts of sunscreen (Nicol, Gaudy, Gouvernet, Richard, & Grob, 2007). A study conducted by Nicol et al. (2007) with 364 beachgoers, showed that the average daily amount of sunscreen used was of 7.67 and 9.33 g/day for the two intervention groups in the study.

An SPF15 sunscreen filters out 93% of UVB radiation, while an SPF30 sunscreen filters out 96%. However, these figures are considerably reduced if less sunscreen than the recommended amount is applied (e.g. only applying half the required amount reduces the protection by two-thirds) (The British Association of Dermatologists, 2013). Moreover, individuals may overestimate the amount of time that they can stay in the sun after applying sunscreen relative to their skin type. Thus individuals might be aware of the sun-protection message, willing to implement and even in the belief that they are protecting themselves, but still be at high risk of considerable sun damage.

Knowledge about skin cancer risk and motivation to prevent sunburns do not seem to play a role on the perceived importance of tanning (Dennis, Lowe, & Snetselaar, 2009). The tanning motive remains popular, in particular in countries with less sunlight exposure such as the UK. The British population receives approximately 30% of their annual UV exposure during their two-week holiday (World Health Organisation, 2002) abroad. Holidaymakers are often seeking a ‘safe tan’, e.g. sufficient sun-exposure to get a visible tan without experiencing burning. To date, there is limited knowledge on how holidaymakers in the UK implement sun-safe behaviours. Only one UK study to date has qualitatively explored the processes involved in the desire to obtain a tanned appearance in holidaymakers. The conclusions were that having a tan is seen as a ‘symbolic artefact’ that usually implies a good holiday (Carter, 1997). The study also showed that participants had a good level of knowledge about negative consequences of sun-exposure, but did not follow the preventive advice for sun-protection. A previous study also explored the desire for a tan amongst young women in England (Lake, Thomson, Twelves, & Davies, 2013) and showed that having a tan is one of the most powerful influence on people’s sun-exposure.

Public health messages for skin cancer prevention in the UK have been in place since 2003 and evidence suggests that holidaymakers do not follow preventive messages and have a strong desire for a tan during holidays. Little is known about how these messages are perceived and implement by individuals during holidays. Information about these perceptions could inform the development of future public health messages targeting sun-protection. This study is unique in considering how these views might impact on experiences of sun-protection during holidays and inform current skin cancer prevention messages.

### Objectives

We conducted an exploratory study with the aim of examining potential gaps in knowledge, motivation and sun-protection practices during holidays, using a combination of semi-structured questions and an objective assessment of sunscreen application. The research questions this paper will answer are: (a) How aware are participants of the current guidelines for sun-protection? (b) What are the main perceived barriers and facilitators for sun-protection? (c) How do participants use sun-protection during holidays? (d) What are the main goals and motivation for using sun-protection during holidays?

## Methods

### Study design

This is a mixed-methods study that combines both qualitative and quantitative information. More specifically, a pragmatic theory-informed approach was used to synthesise the findings from the interviews. The study was reviewed and approved by the Newcastle University.

### Setting

Data was collected between March and May 2013. Participants were recruited from local urban community areas (e.g. supermarkets notice boards, nurseries notice boards, sports groups/associations) and local university. Most invitations to participate were by email or word of mouth.

### Participants

Caucasian participants aged 18 years or older with past experience of sunny holidays abroad were recruited. After replying to the advertisement, potential participants were contacted by the researcher (AR) to provide further information about the study and the participants’ information sheet. Eligible individuals were assessed for inclusion by the researcher (AR) and signed a consent form before participation.

### Data measurement

Participants’ socio-economic status was assessed using the 2015 English index of multiple deprivation (IMD) based on participants’ postcodes of residence. IMD deciles were obtained from the Office for National Statistics (ONS) website (http://www.ons.gov.uk); decile 1 represents the most socio-economically deprived and decile 10 represents the least deprived.

Participants took part in an individual face-to-face interview. At the beginning of the interview, participants answered a standard question about their sun sensitivity based on Fitzpatrick’s skin types (Fitzpatrick, 1988). The Fitzpatrick classification establishes six categories describing the reaction of the skin to the sun: type I – always burns easily, never tans; type II – usually burns easily, tans with difficulty; type III – burns moderately, tans gradually; type IV – rarely burns, always tans well; type V – very rarely burns, tans very easily; type VI – never burns, deeply pigmented. Based on their self-reported skin type, questions about their estimated safe sun-exposure without sun-protection were also included. This information was matched with recommended sun-exposure levels based on skin type – known as personal minimal erythemal dose (UVB MED [mj/cm]). This is an objective measure of sun sensitivity and it specifies the dose of ultraviolet B light required to produce visible redness of the skin (Fitzpatrick, 1988).

Individuals’ knowledge, motivation and experiences of sun-protection during their holidays were assessed in a semi-structured interview guided by a topic guide (see Appendix 1 at online supplemental file at https://doi.org/10.1080/21642850.2017.1335205) based on the ‘theoretical domains framework’^1^ (TDF), using standard methods (Michie et al., 2005). The TDF has been used to explore theory-based factors influencing behavioural change across various health-related behaviours including physical activity during the retirement transition, healthcare practice and adherence to a lifestyle intervention to prevent type 2 diabetes (Francis, O’Connor, & Curran, 2012; McDonald, O’Brien, White, & Sniehotta, 2015; Penn, Dombrowski, Sniehotta, & White, 2013).

Participants were asked about their awareness of specific recommendations for sun-protection and prompted to describe their understanding of it. After this initial question, all participants were shown the relevant guidelines for sun-protection practices by reading a laminated card showing the World Health Organisation (2014) guidelines on how to be sun-safe (see Appendix 2 at online supplemental file at https://doi.org/10.1080/21642850.2017.1335205):

- *Seek shade* when UV rays are the most intense (between 10 a.m. and 4 p.m.),
- *Wear protective clothing* **(**hat with a wide brim, sunglasses, and tightly woven, loose fitting clothes),
- *Use sunscreen.* Apply a broad-spectrum sunscreen of SPF 15+ liberally and re-apply every 2 h, or after working, swimming, playing or exercising outdoors.

The card was used to summarise the definition of sun-protective behaviours and to guide the questions thereafter by acting as a visual cue to what was meant by ‘sun-protective behaviours’ (i.e. card displayed on the table during interview).

Participants were prompted to answer about their intentions to be sun-safe (information displayed in Table 1), these could be positive, negative or ambivalent. Ambivalent intentions would imply some level of incongruence between their intention to be sun-safe in theory and the descriptions and actions on actual sun-safe practices.

**Table 1.** Summary of participants’ characteristics and sunscreen use, ranked by sunscreen quantity (mg).

Participant ID	Age	Gender	Index of multiple deprivation decile	Skin type	Self-reported safe sun-exposure	Recommended sun-exposure UVB MED (mj/cm)	Intention to use sun-protection	Intention to tan	Sunscreen use (mg)/ surface (cm^2^)
Participant 9	24	Female	–	5	Few hours	60–90	Ambivalent	Yes	0.66
Participant 12	43	Male	8	2	20 min	25–40	Yes	No	0.85
Participant 6	46	Female	3	4	60 min	40–60	Ambivalent	Yes	0.88
Participant 14	21	Female	5	3	Few hours	30–50	Yes	No	0.92
Participant 16	49	Female	–	2	20 min	25–40	Yes	No	0.96
Participant 15	28	Male	9	4	20 min	40–60	Yes	Yes	1.13
Participant 1	32	Female	3	3	Few hours	30–50	Yes	No	1.30
Participant 5	35	Female	4	2	30 min	25–40	Yes	Yes	1.30
Participant 8	29	Male	6	3	120 min	30–50	Yes	No	1.34
Participant 4	50	Female	3	3	30–60 min	30–50	Yes	Yes	1.45
Participant 3	30	Female	7	1	5 min	15–30	Yes	No	1.47
Participant 13	55	Female	2	2	30 min	25–40	Yes	No	1.57
Participant 17	26	Female	8	3	Few hours	30–50	Yes	Yes	1.72
Participant 11	23	Female	4	1	30–60 min	15–30	Yes	No	2.07
Participant 10	45	Male	9	2	30 min	25–40	Yes	Yes	2.43
Participant 2	62	Female	10	2	10 min	25–40	Yes	No	3.07
Participant 7	27	Female	2	2	20 min	25–40	Yes	Yes	4.57

Notes: Green line denotes participants above and below the recommended amount of 2 mg/cm^2^, based on a median of 1.34 for sunscreen use.

Interviews were conducted in a room at a university setting that allowed for privacy and confidentiality. All interviews were conducted by a female researcher with experience in interviewing. Special attention was given to assure participants that personalised data collected through interviews would be kept anonymous. Interviews lasted between 30 and 50 min and were audio-recorded with respondent’s consent. The recordings were anonymously transcribed verbatim before analysis. Transcripts were only available to members of the research team. All data from qualitative work were stored anonymously. Identifiers were stored in a protected data file on the computer of a researcher from the team. In line with good governance transcripts will be retained for 10 years so that it is available for reanalysis and audit should this requirement arise.

### Study size

Seventeen participants were recruited an interviewed. We followed standard criteria (Francis et al., 2010) to assess data saturation of interviews.

### Quantitative variables

Participants were also asked to apply sunscreen to their forearm during the interview in the same way they would do it on a sunny day during their holidays (see Additional file 1 for details). The sunscreen bottle was weighed before and after each application (measurement in grams).^2^ Skin surface (where sunscreen was applied) was measured for each participant (Lund & Browder, 1944). To assess other sun-protection practices, the interview included visual aids as prompts for discussion about sun-protection methods (i.e. images with different types of shade, hats and protective clothing).

#### Analytic methods

Transcribed interviews were initially coded using the TDF and thematic analysis (2006) was used to identify overarching themes both within and across the domains using an inductive approach, following a six-phase approach to analysis: (1) familiarisation with the data; (2) generating initial codes; (3) searching for themes; (4) reviewing themes; (5) defining and naming themes and (6) producing a report of the analysis. This enabled the identification of specific themes in the transcripts that provided an explanation of the reported experiences and practices. Interview transcripts were also analysed in separate sub-groups according to participants’ intention to tan and intention to use sun-protection.

In an initial stage, the coding framework was developed by a single researcher (AR) that analysed the transcriptions and generated an initial list of codes. The list was then reviewed, refined and agreed through discussion with team members (AR, FFS, VAS) during data analysis clinics to check for validity, trustworthiness and credibility of the coding and avoid any potential bias in data interpretation.

To facilitate the integration of both qualitative and quantitative levels of analysis, the quantitative data collected during the interviews, such as the objectively assessed sunscreen use and estimated safe sun-exposure can be found in Table 1. To explore sun-protection, observational data on sunscreen use as well as participants’ responses about their self-reported sun-exposure and skin type were analysed alongside narrative accounts.

**Figure 1.**
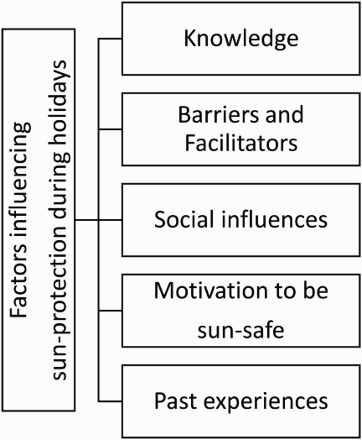
Thematic map of factors influencing sun-protection during holidays.

Quotes have been used to exemplify the themes throughout this paper. Each quote is illustrated with a code that represents participants’ gender, age and skin type (i.e. male, 28, skin type III). Major themes and sub-themes were identified during the analysis by parsimoniously clustering utterances by specific research aims (Figure 1). Findings regarding sub-group analyses according to participants’ intention to use sun-protection and to suntan are also presented.

## Results

### Participants and descriptive data

The sample consisted of 17 individuals (13 female) aged 21–62 years (*M* = 36.8; SD = 11.3) (see Table 1 for participants characteristics). For most participants a sunny holiday comprised of a beach- or pool-side resort and sightseeing. The majority (*n* = 14) were fair-skinned and described their skin as moderately to highly susceptible to burn (skin types 1–3 according to the Fitzpatrick classification (Fitzpatrick, 1988)). The participants’ IMD deciles ranged from 2 to 10 with a median IMD decile of 5.

### Main results

#### Factors influencing sun-protection during holidays

Five main themes were identified from the interviews as factors seen to influence sun-protection during holidays: (a) knowledge, (b) barriers and facilitators, (c) social influences; (d) motivation to be sun-safe and (e) past experiences of sun-protection.

#### Knowledge about sun-protection

While there were some factual gaps in knowledge and some levels of misunderstanding about sun-protection details, participants felt well informed about common sun-safe messages and showed, overall, a good level of awareness.

There was widespread acknowledgement that sun-protection behaviours prevent sunburns, skin damage, skin ageing and skin cancer. Older participants noted that they have become more sun-aware over the years. Participants were also aware of the importance of vitamin D and its association with UVR exposure.

I suppose nowadays because I’m kind of more aware of the health risks erm I’m putting sun cream on to prevent erm you know the risk of skin cancer erm and also to prevent ageing. (male, 28y, skin type IV)

Yes sunburn long term I think the skin, apart from maybe getting sick – you know I think your skin wrinkles more if you spend too much time in the sun. (female, 32y, skin type III)

I guess you get your vitamin D and people feel better when it’s sunny, so … It’s sort of generally uplifting. (female, 23y, skin type II)

Cause I know that a certain proportion in the UK quite a lot of course, are deficient in vitamin D and so I know that I probably, to some degree I need to get more sun. What I get is I get maybe not so much at the moment when I’ve not had money but generally I’d get a burst of sun for a couple of weeks and then no sun for the rest of the year and I’ll be better at trying to … . (male, 28y, skin type IV)

##### Seeking shade from 10 a.m. to 4 p.m.

Participants were conscious of the need for sun-safe behaviours. They also aware of peak UVR hours, however there was a discrepancy from the guidelines, as the majority of interviewees identified peak hours from 11 a.m. to 3 p.m. and some from 12 noon to 2 p.m.

Erm I think knowing that the time of day’s earlier than I thought, like I’d always thought it was something like 12ish. I didn’t realise sort of 10am is still considered peak time for UV rays.. (…) I’m fairly conscious of the fact that it’s sort of from around like erm like midday to 2 o’clock is like sort of like your peak time of that’s when erm you’re going to get burnt and you know it’s prob … that will probably be in some ways too hot for me sometimes so I’ll probably be more conscious of being in the shade then and so yeah, knowing that. (male, 29y, skin type III)

Erm better than being indoors I would probably choose something that tends to be with a tree or an umbrella or something like that. (female, 26y, skin type III)

##### Covering-up strategies

Respondents were aware of the recommendations for covering up through the use of clothes, headwear and sunglasses. Knowledge about clothing was somewhat variable. Some knew about the existence of clothes with a sun-protection factor and the advantages of densely woven clothes with a loose fit and long sleeves. Others, however, described cotton as the textile offering best protection and some were unsure what clothing would offer most protection. Knowledge about covering-up methods was best for hats and sunglasses with most participants displaying good knowledge on how to select appropriate eye and head wear (e.g. wide-brimmed hat).

I sort of wear all the tight woven clothes and broad brimmed hat. (male, 43y, skin type II)

Well I notice this when it is obviously a cotton like top; it is obviously going to protect you more than those kind of beachy ones. I think still some rays pass through there. So I can tell that. (female, 32y, skin type III)

[clothing] I have no idea, I have no idea, me shorts ehm just trying to think of me shorts, me shorts are usually ehm quite protective, because I wear a heavy-ish short, I wear a baseball shorts which is usually come just above the knee. (male, 45y, skin type II)

##### Using sunscreen

Sunscreen was identified by the majority of participants as the first line of defence against the sun. Most participants knew that sunscreen with a SPF 15+ was recommended. There was good awareness for the need to apply sunscreen 30 min before sun-exposure and the importance of taking special care with exposed areas prone to sunburns, often based on personal experiences of burning. Participants knew how often to reapply sunscreen, but a few showed uncertainty about the amount of sunscreen required for optimal protection, noting that the common recommendation to ‘apply sunscreen liberally’ was very vague.

Erm it’s only sun cream that we use (…) I always think of sun cream as like the first, the first thing like first line yeah. (female, 35y, skin type II)

Again it’s something I don’t quite know whether you’re supposed to put it on so you can see the whiteness and let it sink in or you’re supposed to rub it in so that it’s gone. (female, 27y, skin type II)

I’m never quite sure if this is right because I’m not sure but to that it doesn’t matter what level of sun cream you use so say you use a factor 40 or whatever doesn’t change how you tan it just changes the amount of time you can spend out in the sun. That’s the only thing I can remember. (female, 26y, skin type III)

#### Barriers and facilitators of using sun-protection during holidays

##### Seeking shade from 10 a.m. to 4 p.m.

Participants noted, as a barrier for being sun-safe, that availability of shade is often limited in holiday destinations or associated with additional costs (e.g. for hiring a parasol).

I think the only problem is, as I’ve mentioned before, is trying to find shade if you’re on, sort of on the beach. (male, 43y, skin type II)

I suppose you see if the beaches, some beaches have these sort of things and they have umbrellas. If they had yeah more, if more of the hot spots had these sort of things and you didn’t have to pay for them or you could just sit on them. (female, 50y, skin type III)

For some participants implementing sun-safe practices was viewed as interfering with their holiday experience. This was particularly salient when reflecting on seeking shade between 10 a.m. and 4 p.m., perceived by some as a guideline that was too extreme and unhelpful.

You don’t go on a beach holiday to just sit under an umbrella; you go sort of to be in the sun. (male, 29y, skin type III)

What … what … it would be pointless to make, part of the time having lunch, having a nice long lunch or coffees or whatever, we do that quite a lot but I would never ever stay inside between 10 and 4. (…) I wouldn’t … it wouldn’t be worth my while going on holiday. (female, 46y, skin type IV)

Erm well you pay money to sit in the sun and have a bit of heat and obviously get a tan and if you were in to sit in the shade or well you can just think well I can save that money and sit in my garden at home I guess. (female, 27y, skin type II)

Avoiding midday heat was highlighted as facilitator to seek shade during holidays. Forward planning of holiday activities, including opportunities to enjoy shade, was also used as a strategy to be sun-safe.

If it got too hot I’m not averse to moving into the shade really but if you can get one, around lunch time I’m guessing. Erm at lunch I like to sit in the shade. I don’t like to get too hot when I’m eating erm and then I go back to the sun lounger. (female, 27y, skin type II)

Erm well ‘cause I’ve got two young kids I do erm I do a lot of sort of out and about things with the children but sort of midday we tend to be indoors with the children having a nap and things. (female, 35y, skin type II)

If you’re on holiday, you can go to the beach but you know there’s a café or there’s somewhere you can go to for lunch that stops you being just in the sun for the whole day. Where you’d go off and you’d do something else so you’re not just being. (female, 23y, skin type II)

Perceived psychological benefits were also highlighted as a reason to engage in intentional sun-exposure. The fact that feeling the sun on the skin is associated with sensory pleasure was described as an important reason for sun-exposure.

[sun-exposure] Obviously you feel better. Obviously mental health problems, mental health issues. (male, 43y, skin type II)

Well emotionally really as I said I feel better in the sun than I do out of the sun. (female, 46y, skin type IV)

You’ve went outside into the sun and dried off and the sun’s lying there and it was so nice to just lie there after swimming and you’re so relax and feeling the sun. (male, 28y, skin type IV)

##### Covering-up strategies

Concerns about looks and fashion played a significant role in the acceptability of cover-up strategies. The use of hats was sometimes mentioned as conditional to aesthetic and comfort concerns (e.g. looking good). Sunglasses were mostly used to protect eyes from brightness and not for sun-protection. The use of clothes to cover up tended to be related to feeling too hot, rather than to be sun-safe.

Erm as I say I’ll wear a couple because I don’t … sometimes I feel a bit, I’m not really a hat person, I don’t really like wearing hats but I have to so usually erm I would say those two. (…) probably am I not going to look too ridiculous in it. (female, 55y, skin type II)

I would again just really based on style and then probably just t-shirts or thin shirts or thin shorts and yeah. (male, 29y, skin type III)

I don’t do that because then I think well I would get tan lines or won’t get a tan. (female, 24y, skin type V)

##### Using sunscreen

Sunscreen use was seen as a chore involving conscious effort and time. Some described it as something not enjoyable, mentioning specific characteristics of sunscreen (i.e. texture, smell) that make it difficult to use. For some interrupting ongoing activities to reapply sunscreen was viewed as a disturbance to the holiday experience.

It’s just the faff of putting it on, it takes time so that’s mainly it. It’s just the hassle of doing it. (male, 43y, skin type II)

It’s not the nicest thing to put on – it’s quite oily; and doing it at the beach, where’s sand everywhere, it just sticks. (male, 28y, skin type IV)

It’s just such a chore in all honesty. (female, 26y, skin type III)

You’re so relaxed and feeling the sun and there would always have the sensation of well I can’t lie here for too long [without putting more on]. (male, 28y, skin type IV)

The costs associated with sunscreen were also identified as a barrier. Some explicitly suggested that making sunscreen available everywhere would foster its application. Similarly, environmental cues (e.g. sun-safety signs on site) were also mentioned as a way to promote sun-protection behaviours.

Erm I guess if sun cream was cheaper [it would help using more] erm because it is really expensive and therefore especially, especially because then it doesn’t really last. (male, 28y, skin type III)

Well the last few years the hotels and the places we’ve been to have signs up and you know the kids go to kids club and they’ve got to make sure that they’ve got a sun cream on before they go in to visit at the kids club and there’s little signs up everywhere reminding you to sort of things like that. (female, 35y, skin type II)

A reason often given for not engaging in sun-safe practices was ‘forgetting’, especially when considering sunscreen (re)application. Some participants described explicit strategies to overcome forgetting, for example packing sun-protection gear before going on holiday or placing it near the door before in preparation for leaving the holiday apartment to the beach the next morning. The use of sun-protection methods as part of the daily routine (e.g. applying sunscreen after brushing teeth in the morning) was used to prevent forgetting.

Then sometimes I just forget and I’ll just be there and I won’t have anything. (male 28y, skin type III)

I probably put it on in the morning and forget for the rest of the day. (female, 21y, skin type III)

And the sunscreen, yeah and pack them and we usually make sure that we pack enough erm .(female, 23y, skin type I)

If I’m putting sun cream on, I’d usually take a hat with me so I associate them together. (female, 23y, skin type I)

Erm I guess it would be a case of, you know, rather than waiting for it to get hot, like when I’m out, more being conscious of the idea of actually like when I’m leaving the house apply the sun cream then because it’s … it will be around the time that I’d be getting up when I’m on holiday and then going to think of it as a more like, you know, daily routine more like, you know, sort of brushing your teeth almost like, you know, and get ready so yeah. (male, 29y, skin type III)

#### Social influences

When asked about social influences, participants tended to refer to sun-protection in general and described sun-safe behaviours as socially determined. Partners and family were seen as key influences on sun-safe behaviours. In particular, parental influence was perceived as key for developing sun-safe habits.

We go in a group so there’s always, you know, we sort of remind each other type thing so. (female, 35y, skin type II)

She has [partner] certainly got me into wearing sun cream if I’m not wearing. (male, 28y, skin type IV)

Participants admitted that being next to someone who utilises sun-protection behaviours would prompt and remind them to do so too.

I guess if other people are putting sun cream on, that would remind me. If someone’s doing that, – ‘Oh, yeah, I was supposed to. (female, 23y, skin type I)

Nevertheless, social influences were not always seen as facilitating sun-safe behaviour. In some social contexts, the stereotype of someone who regularly avoids direct sunlight is seen as overly cautious instead of someone who is enjoying the moment. Hence, a fear of mockery was expressed by some. Similarly, seeking shade can imply the need to leave social activities when travel companions prefer continuing their activities in the sun.

If you’re on holiday with somebody you wouldn’t want to be everyone in the sun and then me sat by myself not talking to anybody. You wouldn’t really want to have to spend the entire holiday by yourself, which will be happening. (female, 27y, skin type II)

They just like to bake in the sun and they always sort of mock me ‘cause I’m trying to find the shade. (male, 43y, skin type II)

I probably don’t think I’m the coolest of people so I don’t mind spending that extra time putting sun cream on so I don’t get burnt. I see that as an investment. (female, 23y, skin type I)

For some participants, becoming a parent or a grandparent meant being a role model for children and thus, more responsible in terms of their sun-safe behaviours.

With the children it has changed a bit; so I would – before I had the children I would spend quite a long time in the sun. (female, 32y, skin type III)

I’m about to become a grandma I worry more generally about lifestyle behaviours that can have an effect on me not being around for my grandchildren. (female, 46y, skin type IV)

#### Do people intend to be sun-safe during holidays?

When explicitly asked for their motivation, 15 participants expressed a clear intention to use sun-protection during their holidays. Two participants expressed ambivalent intentions and pointed out that sun-protection was not a primary goal during holidays (see Table 1). These two participants did not identify themselves as personally at risk (self-reported skin types IV and V) and still reported some preparatory behaviours, such as buying sunscreen and packing it.

Although all participants expressed some motivation to tan and most, even strong intentions to use sun-safe behaviours, eight interviewees explicitly brought up a motivation to tan during their holidays. This intention was related to positive consideration towards a tanned appearance, and in some cases also towards a social/cultural identity related to tanning prevalent in the North East of England.

I come from that sort of background where I worry about not getting a tan. How on earth would I come back off holiday [without one]. (female, 46y, skin type IV)

Participants also mentioned that having a tan is a symbol of being on holiday, and failing to do so would constitute an opportunity cost.

If I came home from holiday without a tan I’d be gutted (female, 24y, skin type V).

I do kind of go away thinking I don’t want to tan too much so at the end of the holiday I’d be reason – I don’t want to look like I’ve not been on holiday. (male, 28y, akin type III)

I suppose I don’t tan very easily, it would be nice to have, at times you think it would be nice to have a little bit more of a tan when you come back from somewhere. (female, 62y, skin type II)

Erm as wrong as it is the point of going on holiday is to get a nice tan. (female, 46y, skin type IV)

I guess if you go away, which quite often I can do and come back no different in colour people think maybe you haven’t had as good a holiday because you haven’t come back brown. (female, 49y, skin type II)

##### Balancing UVR exposure to tan without getting burnt

About half of the sample explicitly brought up the intention to tan as a key motivation during their holidays. They described that their main goal for the holiday is to find the right balance between getting a tan and using sun-protection. This is achieved by participants trying to get the maximum sun-exposure just short of experiencing sunburns. There was no evidence that participants understood that the tanning of the skin is de facto an indicator of ongoing skin damage even if without experiencing sunburns. From the narratives it was obvious that this strategy might not be overly successful as many participants reported regularly experiencing sunburns during their holidays.

I’m wanting a balance of kind of sun. Some tanning erm but not, you know, unhealthy. (male, 28y, skin type IV)

Erm I usually go for the lowest ‘cause it’s like vanity of trying to tan but also making a token gesture of trying not to be in pain. (male, 29y, skin type III)

Well I have learnt recently that if you stay in the sun shorter hours and still putting sunscreen, eventually for a longish time you still get a bit of a tan. (female, 32y, skin type III)

#### Past experiences of sun-protection

##### Seeking shade from 10 a.m. to 4 p.m.

Most respondents were confident that they would find shade if needed. Seeking shade was, nevertheless, the least used method of sun-protection. If at all, participants reported seeking shade between 12 noon and 2 p.m., mostly linked to lunch breaks. Also, some said that seeking shade was incompatible with their holiday routines (e.g. being ‘out and about’; sightseeing).

(…) I don’t sit in the sun to have my lunch, because it’s too hot for me to eat and sit the sun, so I would obviously seek shade on a lunchtime. (male, 45y, skin type II)

sightseeing] If it’s particularly hot so if it’s kind of like erm southern Mediterranean kind of places then I would disappear for about, it tends to be a bit later doesn’t it so probably from about 12 until 3ish something like that out of the sun, really. (female, 49y, skin type II)

That and, if you are on a holiday where you’re sightseeing and walking round, you can’t do anything. You just put some cream on instead. (female, 21y, skin type III)

As methods of seeking shade, some participants used umbrellas or trees to avoid direct exposure to sunrays. Comfort rather than UVR protection was commonly referred as a reason to seek shade, especially to avoid midday heat.

Yeah. Erm but I don’t tend to lie where the sun is right upon you so I usually lie where there’s a tree or where there’s a hammock or something like that anyway because I get too hot. (female, 26y, skin type III)

##### Covering-up strategies

When participants referred to use cover-up strategies it was mostly a partial protection (i.e. head or eyes by using hat or sunglasses) rather than an extensive body coverage. Using hats was the least used covering-up method.

I use sunglasses but it’s more because I read rather than the sun’s so bad but that’s why I would wear them but erm I don’t know about the clothes and I don’t really wear hats. (female, 24y, skin type V)

##### Using sunscreen

In stark contrast to sun-safe recommendations, participants’ strategy of sun-protection relied almost exclusively on the use of sunscreen. The majority said that they put it on before leaving the house but without taking special care to apply it within any particular time frame (i.e. 30 min before exposure). Participants mentioned applying sunscreen on their most sensitive body parts (e.g. face, shoulders and back) and rubbing it on thoroughly until no white marks are visible.

In fact I would. I would put some on before I left the hotel whereas after the first couple of days I’d probably just put on if I felt a bit of red, a bit of you know heat. (female, 46y, skin type IV)

Erm definitely my … the chest and my shoulders erm and my face. (female, 55y, skin type II)

Reapplying sunscreen seems to be prompted by feeling that the skin was hot or starting to burn and most participants recognise they should be doing it more often than every 2 h, given involvement in distinct activities, but fail to do so.

Erm I’m probably not as rigid to the 2 hours that I should be. I’d probably reapply if I started to feel like it wasn’t protecting anymore which is probably still a too late point so remembering a bit more often I think. (…) A bit like I was starting to burn that’s … then it would just go back on. (female, 27y, skin type II)

Self-reported SPF usage ranged from 10 to 50+. The majority of participants reported using sunscreen with a SPF of 15 or 30 in the first days of their holidays. Some participants acknowledged that they would use the lowest SPF possible so that they could still get a tan. Most participants would change for a lower SPF after approximately one week. This was due to the perception that the skin was more sensitive at the beginning of the holiday. Also, participants would decide for a lower sunscreen if they felt their risk of sunburns was lower and if the skin had started tanning. Some switched to very low SPF tanning oils.

I think probably, at the start of the summer when my skin’s particularly sensitive, I’d probably go for 30 and move down to 20 later in the summer when I seem to have – burn less easily. (female, 23y, skin type II)

If … if we went somewhere and it was 30+ the temperature I’d probably do 30. Probably by the end of the holiday it may have gone down to 15 but erm. (female, 27y, skin type II)

I mean to be honest the [SPF] 2 and the [SPF] 4 I would use to get the colour right as opposed to the protection. (female, 46y, skin type IV)

#### Sunscreen use

Sunscreen use was assessed during the interview by asking participants to actually apply sunscreen to their forearm (Table 1). The objectively assessed sunscreen use test showed the majority of participants (*n* = 12; 70.6%) used less than the recommended quantity of sunscreen (2 mg/cm^2^) with an average of sunscreen application of 1.34 mg/cm^2^. Five participants used less than half of the amount of sunscreen required to achieve the SPF noted on the bottle.

Effective protection from UVR requires individuals to be aware of the time they can safely be exposed to the sun without protection, as this also defines the safe exposure time after using sunscreen.^3^ Safe sun-exposure was also assessed by asking participants about their perceived threshold for a safe sun-exposure. Table 1 compares participants’ self-reported safe sun-exposure with the recommended sun-exposure relative for their skin type. Many participants significantly overestimated the amount of time they could be safely exposed to the sun.

## Discussion

### Key findings

This study aimed to explore sun-safe practices during summer holidays, using a combination of interviews, sun sensitivity questions and the objectively assessed sunscreen use. The findings suggest that, even though participants in this study were well informed about the sun-safe messages, motivated to protect their skin and even perceived themselves as well protected, most participants did not report using effective protective practices to be sun-safe. Similar findings have been shown in recent, larger studies with fair-skinned populations conducted in other European countries (e.g. Austria and France) , where individuals were highly motivated to tan (Haluza, Simic, & Moshammer, 2016), exposed themselves to the sun often (Haluza et al., 2016; Sassolas et al., 2015) and did not use adequate sun-protection methods (Sassolas et al., 2015).

Some gaps in how sun-safe messages were implemented amongst our participants were identified. Firstly, participants’ motivations seemed to be driven by a desire to avoid sunburns by finding the right balance between getting a tan and using sun-protection while on holidays. Secondly, despite the emphasis on UVR avoidance in public health messages, seeking shade was the least reported method of sun-protection and covering-up strategies were mostly implemented as a partial protection (i.e. wearing hats or sunglasses) with inaccurate knowledge of effective protective clothing (e.g. protective textiles). Thirdly, unlike the current guidelines for sun-protection, sunscreen use was described by most participants as the first line of sun-protection. Nevertheless, it is important to acknowledge that most participants used less than the recommended amount needed to achieve the labelled SFP. Adding to this, our findings also suggest people may be overestimating the extent to which they could safely be exposed to the sun without any sun-protection. This is consistent with other studies that have shown how holidaymakers overestimated their sun-protection (Lademann et al., 2004; Petersen, Datta, Philipsen, & Wulf, 2013). The choice for sunscreen was explained as the mode that could lead to the least perceived levels of interference on the holiday experience when comparing to other sun-protection methods. Participants in this study perceived sun-protection as a chore with the potential to remove spontaneity and a carefree lifestyle when on holiday and sunscreen seemed to be viewed as the least disruptive. It might also be the case that the marketing efforts associated with sunscreen make this option more salient than other UVR avoidance methods.

### Limitations and generalisability

This is an exploratory study that provided understanding of the holiday experiences and on how people use sun-protection during holidays. The study provided some evidence on the discrepancies between the way participants use sun-protection and the recommended guidelines. However, the findings should be interpreted in the context of the study limitations. Some caution should be taken in generalising these results given our small sample.

The main strength of this study is the use of a mixed-method approach to collect and analyse data, by combining qualitative information with quantitative and unbiased accounts of how sun-protection is used by the participants. A pragmatic theory-informed approach was used to synthesise the findings from the interviews. The thematic analysis focused on the content of the transcripts and on the identification of specific themes within these. The use of the TDF supported the structure of the topic guide to ensure no relevant potential influences on sun-safe behaviours were missed. However, coding was performed by a single researcher and for this reason inter-rater reliability was not assessed, which can have implications for the trustworthiness of the data and also for the breadth of interpretation.

This study did not aim to estimate how prevalent the issues identified in this research are at a population level. The purpose was instead to bring together a range of motivational and practical issues to explore how people manage risks and identify novel insights. More research is needed to explore the issues identified in this exploratory study in population samples. Here, participants were British holidaymakers aged 21–62, mostly female, with skin types II and III. The British population receives approximately 30% of their annual UV exposure in their two-week holiday (World Health Organisation, 2002), highlighting the relevance of the holiday experience for sun-safe practices in this sample. Given the small sample, it is difficult to draw firm conclusions about patterns based on participant characteristics, and we were not seeking to do so.

The perceptions of participants did not differ significantly from what has been found in previous studies (Garside, Pearson, & Moxham, 2010), but future studies should compare how different holidaymakers are within the UK and/or abroad (e.g. Northern European countries). Even though the sample was mostly comprised of individuals with high levels of awareness of sun-safe messages and good levels of knowledge, given their experiences, beliefs and behaviours, these individuals may be still significantly at risk. It would be relevant to analyse whether these results are replicated in a sample with lower levels of awareness. Participants’ awareness of the nature of the study might have introduced some bias by influencing the content of the respondents’ favourable accounts of sun-protection behaviours and perceptions, although it was emphasised that there were no right or wrong answers. Future studies should aim to recruit more male participants as most studies conducted in this area tend to include mostly female participants (Rodrigues et al., 2013), which can lead to an unbalanced representativeness of gender in the perceptions of sun-protection during holidays.

### Interpretation

Participants seemed motivated to avoid sunburns by trying to achieve an optimal balance between using sun-protection and getting a tan. This is consistent with other studies that have coined the phenomenon known as a ‘non-risk reduction strategy’ (Clarke, Williams, & Arthey, 1997). This can be described as an intention to perform a behaviour (e.g. sun-exposure) until it incites potential negative consequences (e.g. sunburns) whilst still getting the positive effects of this action (e.g. getting a tan). Some of our participants also seemed to overestimate the amount of time that they could safely be exposed to the sun without sun-protection based on their skin type. Similarly, other studies have reported the existence of an optimistic bias (Branstrom, Kristjansson, & Ullén, 2006; Clarke et al., 1997) in sun-protection practices, describing it as a tendency to judge own susceptibility to sunburns as lower than the susceptibility of others, which will lead to less intention to change sun-protection behaviour (Branstrom et al., 2006).

The results from this study suggested the existence of a subtype of those going on holidays: the tan seekers (5 out of 17). In line with previous research showing evidence for this subtype of holidaymakers (O’Riordan, Steffen, Lunde, & Gies, 2008; Pagoto, McChargue, Schneider, & Werth Cook, 2004), our participants placed importance upon get a tan, but reported good intentions to protect. In these studies four categories of beachgoers were identified: (1) low-risk sun worshipper (mostly skin types III and IV); (2) high-risk ‘sunburners’ (mostly skin types I and II); (3) moderate- to high-risk tan seekers (mostly skin types II and III) and (4) low-risk sun indifferent. Both studies (O’Riordan et al., 2008; Pagoto et al., 2004) found that the largest subtype includes holidaymakers with a clear intention to tan, despite having a sensitive skin type that is prone to sunburns. Taken together with our results, these data suggest that public health sun-safe messages may need not only to emphasise the importance of UVR protection, but may also need to focus on *how to* achieve appropriate protection. The present study also adds to our knowledge about existing gaps in sun-safe behaviours awareness and actual practices that may aid shaping future skin cancer prevention messages.

The desire for a tanned appearance and the cultural and social value attributed to a tan was reported by our respondents in line with previous research (Abroms, Jorgensen, Southwell, Geller, & Emmons, 2003; Miles, Waller, Hiom, & Swanston, 2005; Potente, Coppa, Williams, & Engels, 2011). Having a tan was perceived as being healthy, more attractive, as a symbol of being on holiday and spending an enjoyable time abroad. These perceived benefits of having a tan may outweigh the perceived benefits of sun-protection practices and resembles what other studies have also found (Cafri, Thompson, Jacobsen, & Hillhouse, 2009; Garside et al., 2010; Jackson & Aiken, 2000; Lake et al., 2013; Potente et al., 2011). The importance of tanning and culture identity has also been highlighted by studies conducted in Australia with young adults (Leske, Young, White, & Hawkes, 2014).

Environmental resources also emerged as relevant and this is consistent with the findings of a recent systematic review (Rodrigues et al., 2013). Overexposure to the sun was reported to be related to the lack of resources in the environment (e.g. shade availability) or by situational constraints (e.g. concurrent activities like sightseeing) (Garside et al., 2010). The lack of resources could be down to the way the beach was organised (lack of shade, or availability after payment) or could be the making of the person. Several instances of forgetting to engage in relevant preparatory behaviours for sun-protection were reported. Previous studies have also shown the importance of self-regulatory strategies such as forward planning to overcome forgetfulness (Araujo-Soares et al., 2013; Hamilton, Cleary, White, & Hawkes, 2016; Leske et al., 2014).

### Implications for clinicians or policymakers

The findings from this study suggest possible avenues for future research and practice in skin cancer prevention. Strategies to promote sun-safe practices could provide specific instructions on how to perform the behaviours. For instance, while public health messages should make people aware that sunscreen is not the first line of defence against the sun, messages on sunscreen use could provide information on the correct application due to its popularity. Making use of some existent messages, public health campaigns could use real-life quantifiable examples for sunscreen (e.g. for a full body application use at least the equivalent of six full teaspoons (The British Association of Dermatologists, 2013). Messages could also address details about the extent to which people could safely stay in the sun without protection, based on their skin type and SPF used. Public health messages could also emphasise sun-exposure avoidance during peak hours, but framed it as a behaviour that fits the holidaymakers’ routine/lifestyle. For example, seeking shade message could be reframed to a more flexible and realistic time bracket of 12 noon–2 p.m. (e.g. lunch or nap break). In addition, the incorporation of vitamin D and healthy UVR exposure information in sun-protection messages seems to be important. Our participants were aware of the importance of vitamin D and its association with UVR exposure. Webb, Aseem, Kift, Rhodes, and Farrar (2016) highlighted the importance of public health messages to raise awareness about the relationship between vitamin D and sunlight exposure as their study with a culturally diverse sample of 16 British adults reported on the lack of knowledge and confusion about this topic (Webb et al., 2016).

In line with recent studies (Garside et al., 2010; Lake et al., 2013), our participants also placed importance on having a tanned appearance. There is therefore a potential need to address the importance attributed to a tanned appearance, instead of focusing on the damaging effects of sunlight in public health messages. Several studies offer potential strategies to tackle appearance-based concerns, such as promoting sunless tanning as a substitute for sunbathing (Pagoto, Schneider, Oleski, Bodenlos, & Ma, 2010) and observing information about photoaging using UV photographs (Mahler, Kulik, Gibbons, Gerrard, & Harrell, 2003; Rodrigues et al., 2013; Williams, Grogan, Clark-Carter, & Buckley, 2013). Messages could also highlight that sun-exposure leads to skin damage even without sunburns and that any tanning is a sign of UV-induced skin damage (Coelho & Hearing, 2010).

Further research should, for example, explore whether perceptions and beliefs of skin colour change (i.e. what happens from baseline skin colour to red and tanned) and how it relates to personal risk.

## Conclusions

The competing intentions reported by participants suggest that holidaymakers are likely to overexpose to the sun while over-relying on the application of insufficient sunscreen. Public health messages could make people aware of the risks of misapplying sunscreen, by demonstrating a correct application and other effective sun-protection practices.

## Supplementary Material

Supplemental_Data.docx

## Notes

1 The ‘theoretical domains framework’ is a specific approach designed to identify theoretical domains relevant to explain behaviour and behaviour change. These domains can be perceived as barriers or facilitators to behaviour. These 12 domains are: knowledge, nature of behaviour, skills, social/professional role and identity, beliefs about capabilities, beliefs about consequences, motivation and goals, memory, attention and decision processes, environmental context and resources, social influences, emotion and behavioural regulation.

2 In order to estimate sunscreen use, skin surface was estimated using the following formula: skin surface= [(2 × PI × *r* × *H*) + (2 × PI × (*r* × *r*)]). A combination of wrist (*w*) and elbow (*e*) circumferences (*r *= *w* × *e*/2) along with forearm length (*H*) measurement (all in centimetres). In order to account for the surface of the palm of the hand, this formula included an estimation of participant’s hand surface (Lund & Browder, 1944). A ratio was calculated between sunscreen use (converted to milligrams) and skin surface (cm^2^).

3 Multiplying the unprotected sun-exposure time by the SPF number.

## References

[CIT0001] Abroms L., Jorgensen C. M., Southwell B. G., Geller A. C., & Emmons K. M. (2003). Gender differences in young adults’ beliefs about sunscreen use. *Health Education & Behavior*, (1), 29–43. doi: 10.1177/1090198102239257 12564666

[CIT0002] Allom V., Mullan B., & Sebastian J. (2013). Closing the intention–behaviour gap for sunscreen use and sun protection behaviours. *Psychology & Health*, (5), 477–494. doi: 10.1080/08870446.2012.745935 23252669

[CIT0003] Araujo-Soares V., Rodrigues A., Presseau J., & Sniehotta F. F. (2013). Adolescent sunscreen use in springtime: A prospective predictive study informed by a belief elicitation investigation. *Journal of Behavioral Medicine*, (2), 109–123. doi: 10.1007/s10865-012-9415-3 22460361

[CIT0004] Autier P., Mezzetti M., Dore J. F., Monjaud I., Cattaruzza M. S., Renard F., … Osborn J. F. (1998). Sunscreen use, wearing clothes, and number of nevi in 6- to 7-year-old European children. *JNCI Journal of the National Cancer Institute*, (24), 1870–1872. doi: 10.1093/jnci/90.24.1873 9862624

[CIT0005] Branstrom R., Kristjansson S., & Ullén H. (2006). Risk perception, optimistic bias, and readiness to change sun related behaviour. *The European Journal of Public Health*, (5), 492–497. doi: 10.1093/eurpub/cki193 16195355

[CIT0007] Cafri G., Thompson J. K., Jacobsen P., & Hillhouse J. (2009). Investigating the role of appearance-based factors in predicting sunbathing and tanning salon use. *Journal of Behavioral Medicine*, (6), 532–544. doi: 10.1007/s10865-009-9224-5 19653089

[CIT0008] Cancer Research UK (2009a). SunSmart Survey 2003–2008: Significant Trends.

[CIT0009] Cancer Research UK (2009b, 25 September 2009). SunSmart campaign research. Retrieved from http://sunsmart.org.uk/UV-the-sun-and-skin-cancer/how-to-enjoy-the-sun-safely/

[CIT0010] Cancer Research UK (2011, 25 September 2009). SunSmart campaign research. Retrieved from http://www.sunsmart.org.uk/skin-cancer-facts/

[CIT0011] Carter S. (1997). Who wants to be ‘peelie wally’? Glaswegian tourists’ attitudes to sun tans and sun exposure. *Tourism and health: Risks, responses and research.* London: Pinter.

[CIT0012] Centers for Disease Control and Prevention (2012). Sunburn and sun protective behaviors among adults aged 18–29 years – United States, 2000–2010 US. Retrieved from Morbidity and Mortality Weekly Report: http://www.cdc.gov/mmwr/pdf/wk/mm6118.pdf

[CIT0013] Chesnut C., & Kim J. (2012). Is there truly no benefit with sunscreen use and basal cell carcinoma? A critical review of the literature and the application of new sunscreen labeling rules to real-world sunscreen practices. *Journal of Skin Cancer*, (11), 1–4. doi: 10.1155/2012/480985 PMC3357551 22649734

[CIT0014] Clarke V., Williams T., & Arthey S. (1997). Skin type and optimistic bias in relation to the sun protection and suntanning behaviors of young adults. *Journal of Behavioral Medicine*, (2), 207–222. doi: 10.1023/A:1025586829179 9144041

[CIT0015] Coelho S. G., & Hearing V. J. (2010). UVA tanning is involved in the increased incidence of skin cancers in fair-skinned young women. *Pigment Cell & Melanoma Research*, (1), 57–63. doi: 10.1111/j.1755-148X.2009.00656.x 19968819 PMC2810005

[CIT0016] Dennis L. K., Lowe J. B., & Snetselaar L. G. (2009). Tanning behavior among young frequent tanners is related to attitudes and not lack of knowledge about the dangers. *Health Education Journal*, (3), 232–243. doi: 10.1177/0017896909345195 22707763 PMC3374486

[CIT0017] Diffey B. L. (2009). Sunscreens as a preventative measure in melanoma: An evidence-based approach or the precautionary principle? *British Journal of Dermatology*, , 25–27. doi: 10.1111/j.1365-2133.2009.09445.x 19775353

[CIT0018] Ferlay J., Shin H. R., Bray F., Forman D., Mathers C., & Parkin D. M. (2012). *GLOBOCAN 2012 v1.0*. Retrieved from http://globocan.iarc.fr

[CIT0019] Fitzpatrick T. B. (1988). The validity and practicality of sun-reactive skin Type-I through Type-Vi. *Archives of Dermatology*, (6), 869–871. doi: 10.1001/archderm.1988.01670060015008 3377516

[CIT0020] Francis J. J., Johnston M., Robertson C., Glidewell L., Entwistle V., Eccles M. P., & Grimshaw J. M. (2010). What is an adequate sample size? Operationalising data saturation for theory-based interview studies. *Psychology & Health*, (10), 1229–1245. doi: 10.1080/08870440903194015 20204937

[CIT0021] Francis J. J., O’Connor D., & Curran J. (2012). Theories of behaviour change synthesised into a set of theoretical groupings: Introducing a thematic series on the theoretical domains framework. *Implementation Science*, (1), 1–9. doi: 10.1186/1748-5908-7-35 PMC3444902 22531601

[CIT0022] Garside R., Pearson M., & Moxham T. (2010). What influences the uptake of information to prevent skin cancer? A systematic review and synthesis of qualitative research. *Health Education Research*, (1), 162–182. doi: 10.1093/her/cyp060 19858077

[CIT0023] Geller A. C., Cantor M., Miller D. R., Kenausis K., Rosseel K., Rutsch L., … Demierre M. F. (2002). The environmental protection agency’s national sunwise school program: Sun protection education in US schools (1999–2000). *Journal of the American Academy of Dermatology*, (5), 683–689. doi: 10.1067/mjd.2002.121034 12004307

[CIT0024] Geller A. C., Colditz G., Oliveria S., Emmons K., Jorgensen C., Aweh G. N., & Frazier A. L. (2002). Use of sunscreen, sunburning rates, and tanning bed use among more than 10 000 US children and adolescents. *Pediatrics*, (6), 1009–1014. doi: 10.1542/peds.109.6.1009 12042536

[CIT0025] Green A. C., Williams G. M., Logan V., & Strutton G. M. (2011). Reduced melanoma after regular sunscreen use: Randomized trial follow-up. *Journal of Clinical Oncology*, (3), 257–263. doi: 10.1200/JCO.2010.28.7078 21135266

[CIT0026] Haluza D., Simic S., & Moshammer H. (2016). Sun exposure prevalence and associated skin health habits: Results from the Austrian population-based UVSkinRisk survey. *International Journal of Environmental Research and Public Health*, (1), 141. doi: 10.3390/ijerph13010141 PMC4730532 26797627

[CIT0027] Hamilton K., Cleary C., White K. M., & Hawkes A. L. (2016). Keeping kids sun safe: Exploring parents’ beliefs about their young child’s sun-protective behaviours. *Psycho-Oncology*, (2), 158–163. doi: 10.1002/pon.3888 26101815

[CIT0028] Heckman C., & Coups E. (2011). Correlates of sunscreen use among high school students: A cross-sectional survey. *BMC Public Health*, (1), 679. doi: 10.1186/1471-2458-11-679 21884577 PMC3179453

[CIT0029] Jackson K. M., & Aiken L. S. (2000). A psychosocial model of sun protection and sunbathing in young women: The impact of health beliefs, attitudes, norms, and self-efficacy for sun protection. *Health Psychology*, (5), 469–478. doi: 10.1037/0278-6133.19.5.469 11007155

[CIT0030] Lademann J., Schanzer S., Richter H., Pelchrzim R. V., Zastrow L., Golz K., & Sterry W. (2004). Sunscreen application at the beach. *Journal of Cosmetic Dermatology*, (2), 62–68. doi: 10.1111/j.1473-2130.2004.00107.x 17147557

[CIT0031] Lake J. R., Thomson C. S., Twelves C. J., & Davies E. A. (2013). A qualitative investigation of the motivations, experiences and views of female sunbed users under the age of 18 in England. *Journal of Public Health*. doi:10.1093/pubmed/fds107%U http://jpubhealth.oxfordjournals.org/content/early/2013/01/17/pubmed.fds107.abstract 23365261

[CIT0032] Lazovich D., Vogel R. I., Berwick M., Weinstock M. A., Warshaw E. M., & Anderson K. E. (2011). Melanoma risk in relation to use of sunscreen or other sun protection methods. *Cancer Epidemiology Biomarkers & Prevention*, (12), 2583–2593. doi: 10.1158/1055-9965.EPI-11-0705 PMC4399380 22016471

[CIT0033] Leske S., Young R. M., White K. M., & Hawkes A. L. (2014). A qualitative exploration of sun safety beliefs among Australian adults. *Australian Psychologist*, (4), 253–270. doi: 10.1111/ap.12054

[CIT0034] Linos E., Keiser E., Fu T., Colditz G., Chen S., & Tang J. (2011). Hat, shade, long sleeves, or sunscreen? Rethinking US sun protection messages based on their relative effectiveness. *Cancer Causes & Control*, (7), 1067–1071. doi: 10.1007/s10552-011-9780-1 21637987 PMC3873510

[CIT0035] Lund C., & Browder N. (1944). The estimation of areas of burns. *Surg Gynecol Obstet*, , 352–358.

[CIT0036] Mahler H. I., Kulik J. A., Gibbons F. X., Gerrard M., & Harrell J. (2003). Effects of appearance-based interventions on sun protection intentions and self-reported behaviors. *Health Psychology*, (2), 199–209. doi: 10.1037/0278-6133.22.2.199 12683740

[CIT0037] McDonald S., O’Brien N., White M., & Sniehotta F. F. (2015). Changes in physical activity during the retirement transition: a theory-based, qualitative interview study. *International Journal of Behavioral Nutrition and Physical Activity*, (1), 1–12. doi: 10.1186/s12966-015-0186-4 25889481 PMC4343052

[CIT0038] Michie S., Johnston M., Abraham C., Lawton R., Parker D., & Walker A. (2005). Making psychological theory useful for implementing evidence based practice: a consensus approach. *Quality and Safety in Health Care*, (1), 26–33. doi: 10.1136/qshc.2004.011155 15692000 PMC1743963

[CIT0039] Miles A., Waller J., Hiom S., & Swanston D. (2005). SunSmart? Skin cancer knowledge and preventive behaviour in a British population representative sample. *Health Education Research*, (5), 579–585. doi: 10.1093/her/cyh010 15644381 PMC3943395

[CIT0040] Montague M., Borland R., & Sinclair C. (2001). Slip! Slop! Slap! and SunSmart, 1980–2000: Skin cancer control and 20 years of population-based campaigning. *Health Education & Behavior*, (3), 290–305. doi: 10.1177/109019810102800304 11380050

[CIT0041] National Institute for Health and Clinical Excellence (NICE) (2011a). National campaigns (UK and worldwide). *Expert paper*.

[CIT0042] National Institute for Health and Clinical Excellence (NICE) (2011b). *Skin cancer: Prevention using public information, sun protection resources and changes to the environment*. (32) London: NICE Public Health Guidance.

[CIT0043] Nicol I., Gaudy C., Gouvernet J., Richard M. A., & Grob J. J. (2007). Skin protection by sunscreens is improved by explicit labeling and providing free sunscreen. *Journal of Investigative Dermatology*, (1), 41–48. doi: 10.1038/sj.jid.5700509 17068486

[CIT0044] O’Riordan D. L., Steffen A. D., Lunde K. B., & Gies P. (2008). A day at the beach while on tropical vacation: Sun protection practices in a high-risk setting for UV radiation exposure. *Archives of Dermatology*, (11), 1449–1455. doi: 10.1001/archderm.144.11.1449 19015419

[CIT0045] Pagoto S. L., McChargue D. E., Schneider K., & Werth Cook J. (2004). Sun protection motivational stages and behavior: Skin cancer risk profiles. *American Journal of Health Behavior*, (6), 531–541. doi: 10.5993/AJHB.28.6.6 15569587

[CIT0046] Pagoto S. L., Schneider K. L., Oleski J., Bodenlos J. S., & Ma Y. (2010). The sunless study: A beach randomized trial of a skin cancer prevention intervention promoting sunless tanning. *Archives of Dermatology*, (9), 979–984. doi: 10.1001/archdermatol.2010.203 20855696 PMC3221310

[CIT0047] Parkin D. M., Mesher D., & Sasieni P. (2011). Cancers attributable to solar (ultraviolet) radiation exposure in the UK in 2010. *British Journal of Cancer*, (S2), S66–S69. doi: 10.1038/bjc.2011.486 22158324 PMC3252056

[CIT0048] Penn L., Dombrowski S. U., Sniehotta F. F., & White M. (2013). Participants’ perspectives on making and maintaining behavioural changes in a lifestyle intervention for type 2 diabetes prevention: A qualitative study using the theory domain framework. *BMJ Open*, (6), e002949. doi: 10.1136/bmjopen-2013-002949 PMC3696871 23811173

[CIT0049] Petersen B., Datta P., Philipsen P. A., & Wulf H. C. (2013). Sunscreen use and failures – on site observations on a sun-holiday. *Photochemical & Photobiological Sciences*, (1), 190–196. doi: 10.1039/C2PP25127B 23023728

[CIT0050] Potente S., Coppa K., Williams A., & Engels R. (2011). Legally brown: using ethnographic methods to understand sun protection attitudes and behaviours among young Australians ‘I didn’t mean to get burnt – it just happened!’. *Health Education Research*, (1), 39–52. doi: 10.1093/her/cyq066 21059798

[CIT0051] Rodrigues A., Sniehotta F. F., & Araujo-Soares V. (2013). Are interventions to promote sun-protective behaviors in recreational and tourist settings effective? A systematic review with meta-analysis and moderator analysis. *Annals of Behavioral Medicine*, (2), 224–238. doi: 10.1007/s12160-012-9444-8 23229160

[CIT0052] Rogers R. W. (1975). A protection motivation theory of fear appeals and attitude change1. *The Journal of Psychology: Interdisciplinary and Applied*, (1), 93–114. doi: 10.1080/00223980.1975.9915803 28136248

[CIT0053] Saraiya M., Glanz K., Briss P. A., Nichols P., White C., Das D., … . Rochester P. (2004). Interventions to prevent skin cancer by reducing exposure to ultraviolet radiation: A systematic review. *American Journal of Preventive Medicine*, (5), 422–466. 15556744 10.1016/j.amepre.2004.08.009

[CIT0054] Sassolas B., Grange F., Touboul C., Lebbe C., Saiag P., Mortier L., … . Robert C. (2015). Sun exposure profile in the French population. Results of the EDIFICE Melanoma survey. *Journal of the European Academy of Dermatology and Venereology*, , 6–10. doi: 10.1111/jdv.12895 25639926

[CIT0055] Schwarzer R. (1992). Self-efficacy in the adoption and maintenance of health behaviors: Theoretical approaches and a new model. In *Self-efficacy: Thought control of action* (pp. 217–243). Washington, DC: Hemisphere.

[CIT0056] Sinclair C., & Foley P. (2009). Skin cancer prevention in Australia. *British Journal of Dermatology*, , 116–123. doi: 10.1111/j.1365-2133.2009.09459.x 19775367

[CIT0057] The British Association of Dermatologists (2013). *Sunscreen factsheet*. Retrieved from http://www.bad.org.uk/desktopDefault.aspx?TabId = 734

[CIT0058] Webb A. R., Aseem S., Kift R. C., Rhodes L. E., & Farrar M. D. (2016). Target the message: A qualitative study exploring knowledge and cultural attitudes to sunlight and vitamin D in Greater Manchester, UK. *British Journal of Dermatology*, (6), 1401–1403. doi: 10.1111/bjd.14800 27292412

[CIT0059] Weinstein N. D. (1988). The precaution adoption process. *Health Psychology*, (4), 355–386. doi: 10.1037/0278-6133.7.4.355 3049068

[CIT0060] Williams A. L., Grogan S., Clark-Carter D., & Buckley E. (2013). Appearance-based interventions to reduce ultraviolet exposure and/or increase sun protection intentions and behaviours: A systematic review and meta-analyses. *British Journal of Health Psychology*, (1), 182–217. doi: 10.1111/j.2044-8287.2012.02089.x 22989352

[CIT0061] World Health Organisation (2002). Sun protection: An essential element of health-promoting schools. *WHO information series on school health*.

[CIT0062] World Health Organisation (2014). *INTERSUN Programme*. Retrieved from http://www.who.int/uv/intersunprogramme/en/

