# Supplementary material for: Aware, motivated and striving for a ‘safe tan’: an exploratory mixed-method study of sun-protection during holidays
Source: Health Psychol Behav Med. 2017 Jun 5;5(1):276–98. doi: 10.1080/21642850.2017.1335205 (PMC5470104; doi:10.1080/21642850.2017.1335205)

**Appendix 1**

**Topic guide for interviews**

**Introduction**

**The purpose of this study is: 1) to understand perceptions of holidaymakers about protecting the skin from the sun; and 2) to understand how they enjoy their holidays. In this interview, we would like you to: 1) share your thoughts on protecting your skin from the sun while on holidays; and 2) provide feedback on a new mobile phone intervention that could support holidaymakers in protecting their skin.**

**There are no right or wrong answers to the questions. Take your time to answer each question and, if you prefer, take a few minutes to think about it before answering.**

1. Skin assessment:

Which of the following best describes your reaction to an initial sun exposure of 45-60 minutes (without sun protection) around midday in the early UK summer?

1. Burn easily, never tan
2. Burn easily, tan minimally with difficulty
3. Burn moderately, tan moderately
4. Burn minimally, tan moderately and easily
5. Rarely burn, tan profusely
6. Never burn, tan profusely

2. Opening question:

How would you describe a typical day during your holidays? (Prompt for schedule during morning, afternoon, evening; typical clothes you use on the beach; what you usually take with you to the beach; if you take mobile phone with you to the beach)

3. Knowledge

**3.1** Considering your skin type, how much time do you think you can spend exposed to the sun without sun protection?

**3.2** Do you know of any methods for sun protection?

**3.3** Are you aware of the recommendations for sun protection? (Prompt for specific knowledge of these recommendations (specify SPB based).

**3.4** What time of the day do you think sun protection is most needed? (Prompt for cloudy day)

If they don’t know, please show the laminated card mentioning sun-protective measures according to WHO (see Appendix 2).

- **Seek shade** when UV rays are the most intense (between 10am to 4pm),
- **Wear protective clothing (** hat with a wide brim, sunglasses, and tightly woven, loose fitting clothes),
- **Use sunscreen.** Apply a broad-spectrum sunscreen of SPF 15+ liberally and re-apply every two hours, or after working, swimming, playing or exercising outdoors.

[Use the card to summarize the definition of sun-protective behaviours and as a visual cue to guide the questions from then on. Display card on the table during interview).

4. Nature of behaviours

**4.1** In terms of aiming to improve [specify behaviours]:

. What do you think you might need to do differently?

. What would you do differently, when, where, how, how often and with whom?

**4.2** Can the context be used to prompt these behaviours?

**4.3** How do you know whether the behaviour has happened? (Prompt to sunburn)

5. Skills

**5.1** Do you know how to apply sunscreen? (Prompt for quantity, where to apply (body parts), and how much time before sun exposure)

**5.2** What is the sunscreen SPF that you usually use?

**5.2** How easy or difficult would it be for you to apply sunscreen?

**5.3** Could you please cream your forearm? [Sunscreen bottle will be weighted before and after procedure]

**5.4** Do you know how to choose from different types of [shade/protective clothes/hat/sunglasses]?

**5.5** [Various types of hats will be shown to participants] which of these hats is similar to the one you usually use?

6. Social influences (norms)

**6.1** What would your family and friends think of you using [specify behaviours]?

. What do you think their views might be?

. How might the views of your family and friends affect you doing [specify behaviours]?

7. Social/professional role and identity

**7.1** Do you think these behaviours [show card again] are compatible with your identity/personality (i.e. way your view yourself) (Prompt to different roles that may influence: parent, professional, friend)?

8. Beliefs about capabilities

**8.1** How confident are you about doing [specify behaviours]?

**8.2** What problems do you think you might encounter in doing [specify behaviours]?

**8.3** What would help you to overcome these problems?

**8.4** What would make it easier for you?

9. Beliefs about consequences

**9.1** What do you think would happen if you do [specify behaviour]? (Prompt for positive/negative, long/short term consequences, e.g. : vitamin D issues, physical comfort/discomfort of sunscreen)

**9.2** What are the costs of [specify behaviour]?

**9.3** Do benefits of doing [specify behaviours] outweigh the costs?

**9.4** What do you think will happen if you don’t do [specify behaviours]?

**9.5** How would you feel if you don’t do [specify behaviours]?

10. Motivation and goals (intention)

**10.1** How much do you want to do [specify behaviour?]

**10.2** Does performing [specify behaviours] conflict/interfere with any of the other goals you might have for your holiday?

11. Memory, attention and decision processes

**11.1** What are your reasons for not doing [specify behaviour] during your holiday (prompt for forgetting, keeping track on time, competing activities, etc.)?

Possible questions to prompt further information:

. Will you need to think to do [specify behaviour]?

. How much attention will you have to pay to keep track of time for sunscreen use?

. Will you remember to do [specify behaviour]? What strategies do you use, if any, to remember?

12. Environmental context and resources

**12.1** To what extent do other factors help/stop you from [specify behaviours] (prompt for shade availability, store nearby, UV display or information, money)?

13. Emotion

**13.1** How do you feel about spending time in the shade from 10am to 4 pm while on holiday?

14. Behavioural regulation

**14.1** How would you organise your holiday to [specify behaviours]? For example, would you plan ahead or have any set routines?

**14.2** Have you found any ways of helping yourself remember to do [specify behaviour]. If yes, what ways have you used?

**APPENDIX 2:**


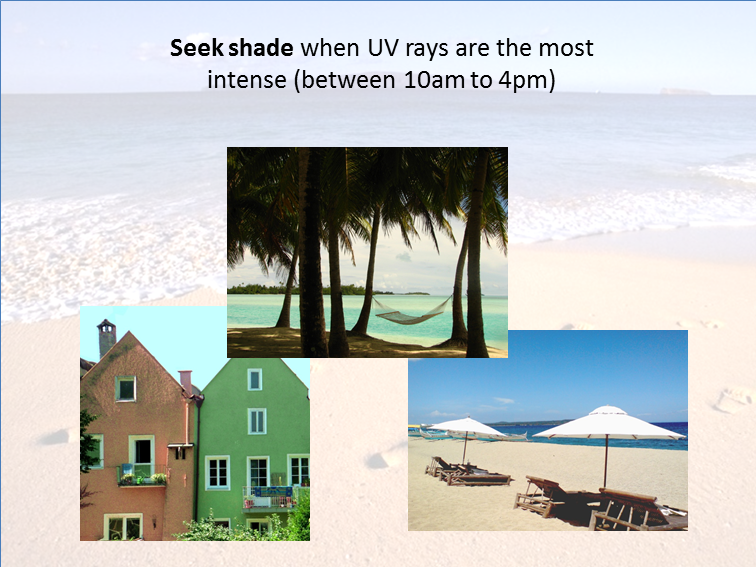


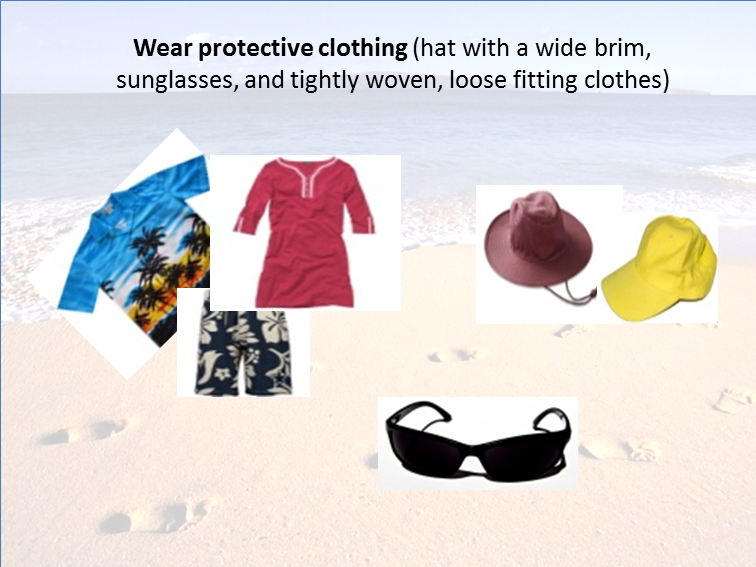

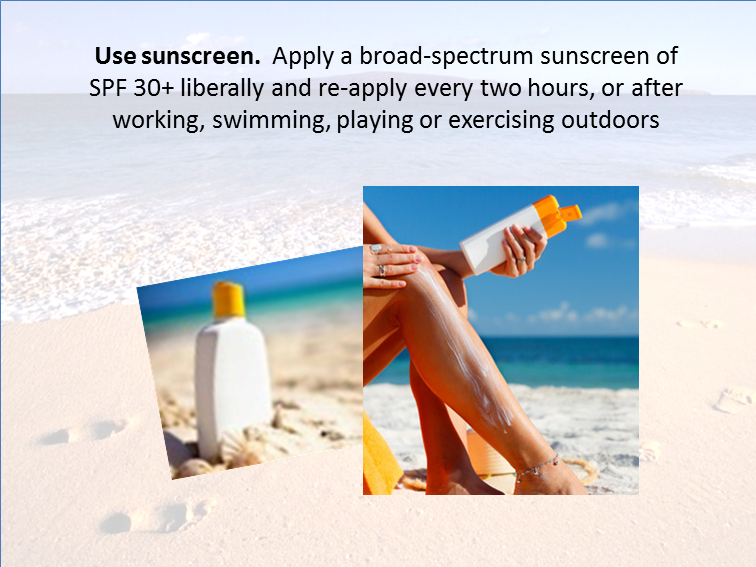

Supplement: Supplemental_Data.docx [file rhpb_a_1335205_sm4006.docx]
